# 

**Published:** 1916-10-28

**Authors:** 


### PASSING EVENTS AND TOPICS.

### Red Cross Needlework Exhibition.

An exhibition of needlework, with a special class for wounded soldiers, will be held at the Central Hall, Westminster, from November 7 to 10, when the *Daily Sketch* is offering £1,000 in prizes. The Marchioness of Ripon opens the exhibition on the first day, and the entire proceeds, free of all expense, will be given to the Red Cross.

### A Woman Splint Consultant.

The talent displayed by women in the organisation and running of hospital supply depôts has been remarkable. At the American Ambulance situated at Neuilly, Paris, an American lady, who is in charge of the splint department, has organised a splint factory, where a large number of splints of great variety are turned out. She has developed such talent for this particular kind of work that she has several times visited by special request many of the hospitals and dressing stations at the Front, or given advice about the application of splints and suggestions regarding new ones.

### Founders' Day at the National Hospital.

The National Hospital for the Paralysed and Epileptic, Queen Square, W.C., is following the example of the Middlesex Hospital and other institutions in arranging a special day when friends and supporters may visit the hospital and inspect the arrangements made for the treatment and comfort of the patients. November 2 is the anniversary of the foundation of the National Hospital in 1859, and invitations have been issued to a short service of thanksgiving in the chapel at 3 p.m., and an inspection of the military and civilian wards and other features of the work.

### Gifts to Hospitals.

Under the will of Mrs. Sarah Laura Sophia Geldart, widow of the late Mr. Henry Charles Geldart, late of Malden House, Huntingdon, Lincoln County Hospital receives a bequest of £1,500.

The Royal Infirmary, Edinburgh, has received notice of the following legacies: Mr. A. S. Douglas, £2,000 and one-fifth of the residue of his estate, and £1,000 to the Convalescent Home; Miss Mary Hamilton, £7,500; Mr. Robert Wilson, £100.

The sum of £1,000 has been collected through Mr. T. R. St. Johnston, the District Commissioner of the Lau Islands, for the purchase of a pair of the latest type of Red Cross motor ambulances for the Front. The Lau Islands are a group lying about 200 miles to the east of Fiji; and the natives, an intellectual type of the Polynesian race, are intensely loyal, and have previously subscribed largely to various patriotic funds. The islands produce about £100,000 worth of the all-important copra per annum.

### Matrons' and Sisters' Appointments.

Cranleigh School.—Miss S. A. Musson has been appointed head matron. She was trained at Liverpool Royal Infirmary, and has since been ward sister and night superintendent at Hull Royal Infirmary, sister-in-charge of the enteric wards at Hull Corporation Sanatorium, night sister at the Royal Hospital for Diseases of the Chest, City Road, E.C., assistant matron at the West London Hospital, and matron of King William's College, Isle of Man, and of Loretto School, Musselburgh.

Mount Vernon Hospital, Northwood.—Miss Priscilla Sanderson has been appointed matron. She was trained at the Middlesex Hospital, and has since been day sister, night superintendent, assistant matron, and home sister at the Royal National Hospital for Consumption, Ventnor.

Lincoln County Hospital.—Miss Edith M. Yeedon has been appointed sister of the military ward. She was trained at the Royal Portsmouth Hospital, and has since been sister at the Kent and Canterbury Hospital.

Salford Union Infirmary, Pendleton.—Miss Ethel Fairclough and Miss Hannah Bocock have been appointed charge sisters. Miss Fairclough was trained at Ecclesall Infirmary, Nether Edge, where she was later temporary sister, and Miss Bocock was trained at Woolwich Infirmary, and has since been a Queen's Nurse at Manchester.

Bowling Park Institution, Bradford.—Miss Bessie Young has been appointed sister and Miss E. G. Holmes maternity sister. Miss Young received her training at Townley's Hospital, Bolton, where she was later charge nurse, night sister, home sister, and assistant superintendent nurse, and has also done private and district nursing. Miss Holmes was trained at Ecclesall Infirmary, where she became sister. She holds the certificate of the Medico-Psychological Society.

Leeds, Township Infirmary, Beckett Street.—Miss Mary Wilkinson and Miss M. Iddenden have been appointed ward sisters. The former was trained at this infirmary, and has since been staff nurse at the Military Hospital, Gosforth, Newcastle-on-Tyne. Miss Iddenden was trained at Ecclesall Infirmary, Sheffield, and has since been staff nurse. She holds the C.M.B. certificate.

**NOTE.—Particulars of Appointments for insertion in this column will be welcomed.**

### The Book Market.

LIST OF NEW BOOKS RECEIVED FROM THE FOLLOWING PUBLISHERS:—

Geo. Allen and Unwin, Ltd.: "Fecundity *versus* Civilisation." By Adelyne More. 6d. net.

Cassell and Co.: "Pulmonary Tuberculosis in General Practice." By Halliday Sutherland. 10s. 6d. net.

Sampson Low and Co.: "The Chronicles of the Imp." By Jeffery Farnol. 2s. net.

G. P. Putnam's Sons: "Materia Medica for Nurses." By Lavina Dock. 5s. net.

### Papers, Pamphlets, etc.

Report of the Committee on Public Health and National Quarantine, United States Senate, on S. 4086, a Bill to Provide for the Care and Treatment of Persons Afflicted with Leprosy, and to Prevent the Spread of Leprosy in the United States. (Washington Government Printing Office. 1916.)

Leprosy as a National and International Problem. By Frederick L. Hoffman, LL.D. Reproduced from the *Journal of Sociologic Medicine.*

RATES OF SUBSCRIPTION: Twelve months. 6/6; Six months, 4/-; Three months, 2/-; post free to any address in the United Kingdom. Abroad, Twelve months, 8/8; Six months, 5/-; Three months, 2/6, post free. THE HOSPITAL "Index" to Volume LX., April to September 1916, is now ready, price 1½d. per copy, post free. Address The Manager, "THE HOSPITAL," The Hospital Building, 28/29 Southampton Street, Strand, London, W.C.

